# Application of Artificial Neural Networks to a Model of a Helicopter Rotor Blade for Damage Identification in Realistic Load Conditions

**DOI:** 10.3390/s24165411

**Published:** 2024-08-21

**Authors:** Pietro Ballarin, Giuseppe Sala, Marco Macchi, Irene Roda, Andrea Baldi, Alessandro Airoldi

**Affiliations:** 1 Department of Aerospace Science and Technology, Politecnico di Milano, 20156 Milan, Italy; giuseppe.sala@polimi.it (G.S.); alessandro.airoldi@polimi.it (A.A.); 2 Department of Management, Economics and Industrial Engineering, Politecnico di Milano, 20156 Milan, Italy; marco.macchi@polimi.it (M.M.); irene.roda@polimi.it (I.R.); 3 Leonardo S.p.A., Helicopters Division, 21017 Cascina Costa di Samarate, Italy; andrea.baldi01@leonardo.com

**Keywords:** fiber bragg grating sensors, structural health monitoring, artificial neural network, composite structure, rotor blade, load monitoring

## Abstract

Monitoring the integrity of aeronautical structures is fundamental for safety. Structural Health Monitoring Systems (SHMSs) perform real-time monitoring functions, but their performance must be carefully assessed. This is typically done by introducing artificial damages to the components; however, such a procedure requires the production and testing of a large number of structural elements. In this work, the damage detection performance of a strain-based SHMS was evaluated on a composite helicopter rotor blade root, exploiting a Finite Element (FE) model of the component. The SHMS monitored the bonding between the central core and the surrounding antitorsional layer. A damage detection algorithm was trained through FE analyses. The effects of the load’s variability and of the damage were decoupled by including a load recognition step in the algorithm, which was accomplished either with an Artificial Neural Network (ANN) or a calibration matrix. Anomaly detection, damage assessment, and localization were performed by using an ANN. The results showed a higher load identification and anomaly detection accuracy using an ANN for the load recognition, and the load set was recognized with a satisfactory accuracy, even in damaged blades. This case study was focused on a real-world subcomponent with complex geometrical features and realistic load conditions, which was not investigated in the literature and provided a promising approach to estimate the performance of a strain-based SHMS.

## 1. Introduction

This work was focused on the monitoring of a critical component, represented by a composite helicopter rotor blade root, which requires complex and costly inspection techniques. In the past, a feasibility study was carried out [1] that was aimed at assessing the technology to embed optical fiber sensors in composite rotor blades. For this reason, the scope of this work was to investigate the design methodologies and the potential performances of an optical fiber-based Structural Health Monitoring System (SHMS) that was applied to a composite rotor blade root as a completion of the technological activities carried out in the past [1]. In the continuation of this introduction, some aspects related to the optical fiber sensors, the criticalities of adhesive bondings, and the algorithms needed to identify the potential damage were discussed.

Composite materials are widely used in the aerospace field since they exhibit good performances in terms of high strength, stiffness, and low density. Optimal fatigue performance in tension and the possibility of producing large monolithic parts without junctions represent other appealing aspects motivating the adoption of composite materials for primary structural parts. Composite structures usually require complex manufacturing procedures and a high manpower. The complexity of such manufacturing processes can give rise to manufacturing defects [2,3,4], which can propagate in the structure during its operational life [5,6,7], affecting the structural properties. Defects can be related to both the fibers and matrix. In the first case, the instability of the fibers creates wrinkles during the lamination phase [8,9]. In the second case, voids can nucleate in the matrix during the curing cycle or can be trapped during the lamination [10,11]. Bondings are also subjected to manufacturing defects like porosity [12,13], but also faulty curing and contamination with release agents, de-icing fluid, or fingerprints can occur [14]. Such defects affect properties like adhesive strength and toughness up to a degradation below 50% [15,16]. In some cases, it can happen that during the polymerization no chemical interaction occurs between the adhesive and the bonded surfaces: this is known as a kissing bond, and in this case, conventional Non Destructive Techniques (NDTs) cannot detect these defects because material continuity is guaranteed. The measurement of local vibration nonlinearity and acoustic microscopy demonstrated good detection performances [17,18] for this purpose. NDTs can help detect defects or damages inside composite components [19,20,21] but they have some drawbacks. First, they are able to inspect the structure only locally, and second, usually the component must be disassembled from the aircraft’s structure to be inspected, increasing the aircraft’s downtime with the consequent loss of revenue. SHMSs, differently from NDTs, provide an on-line inspection of the structure without the need to disassemble structural components, and they are able to monitor the structure in a more diffuse area [22]. Nowadays, different types of SHMSs are studied, such as the ones based on piezoelectric wafer active sensors, piezoresitive sensors, fiber Bragg grating (FBG) sensors, and the ones based on comparative vacuum [23,24,25]. Overall, FBG-based SHMSs seem to be particularly promising for the monitoring of composite structures as they can be embedded and protected inside composite laminates [26], and the sensors can be used to monitor the curing cycle [27,28]. Moreover, they are not affected by electromagnetic disturbances, and they are characterized by a reduced weight [29,30,31,32]. FBG sensors are characterized by some gratings inscribed inside the core of the optical fiber by means of UV radiations. Gratings are formed by periodically changing the refractive index in a small portion of the optical fiber. When the light passes through the grating, part of the light spectrum is reflected, depending on the period of the grating, and this feature is exploited for the strain sensing. Over the years, different techniques were developed to integrate FBG sensors into composite laminates: they can be bonded over the laminate’s surface using some sensorized ribbons, or they can be embedded inside the laminate [33]. The former technique is easier; however, it has some drawbacks related to the sinking of the ribbon in the laminate during the curing phase. The latter technique allows a better protection of the sensors despite its higher complexity.

Many studies were presented in the literature regarding the implementation of SHMSs using machine-learning tools [34,35]. For instance, in [36], the authors identified a damaged welded structure by adopting an Artificial Neural Network (ANN) simulating a pristine lamb wave signal, which was compared with the measured signal to detect the damage. In [37,38], the performance of an SHMS based on an ANN for damage detection was evaluated in a metallic, stiffened helicopter panel. In [39], a methodology for damage detection in aerospace structures that was based on the Gaussian process was proposed. In [40,41], a methodology for damage detection under variable loads was presented and applied to a UAV composite wing. In [42], the Convolutional Neural Networks (CNNs) were adopted for damage detection in the same type of structure.

The above-mentioned criticalities regarding the adhesive bondings, the potential of the machine learning techniques, and the lack of research regarding the damage detection performance on the connection between the blade and the rotor hub motivated the study presented in this work. In fact, the geometrical complexity of the subcomponent, coupled with the complexity of the loading scenario, may severely affect the damage detection capability of an SHMS. This study demonstrates how a Finite Element (FE) model of the subcomponent can be exploited to define a procedure to train a load identification and damage detection algorithm and to assess the performance of the SHMS, thus resulting in a fundamental tool in the SHMS’s design. This approach was applied to a helicopter blade root, which represents a critical subcomponent of the blade, as it shows a high manufacturing complexity, thus increasing the probability to include manufacturing defects. An experimentally validated FE model of the component was exploited to train an ANN-based algorithm. Three versions of the algorithm were proposed, with the aim to detect damages in the adhesive layer joining the central core and the external antitorsional layer of the blade root. These versions adopted different strategies to recognize the applied loads: such a step was needed to uncouple the variability in the strain field due to damages from the one inherent to the variation of the applied loads. This work was divided into four parts: in Section 2, the component under analysis was described, as well as its modeling with FE and the model validation; in Section 3, the three versions of the damage detection algorithm were presented, together with the methodology for the creation of the dataset to train the ANN; in Section 4, the performances of the three versions of the algorithm were investigated under different levels of noise; finally, in Section 5, the conclusions and main findings were discussed.

## 2. The Description and Modeling of the Blade Root

### 2.1. Blade Root Description

**Figure 1:**
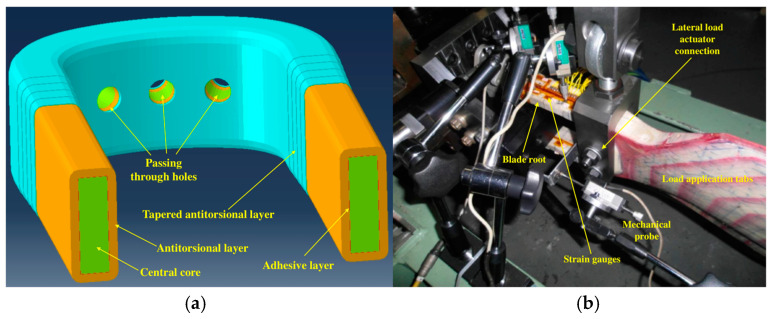
Pictures describing the blade root: (**a**) blade root with details including its internal features; (**b**) blade root subjected to testing in the test rig.

The component was represented by a composite helicopter rotor blade root, which is shown with the details including the internal features in Figure 1a, while in Figure 1b, the blade root subjected to testing can be observed. Referring to Figure 1a, the following colors were used to distinguish the main subcomponents: the color light green was used for the central core, the color orange was used for the antitorsional layer, the color light blue was used for the tapered antitorsional layer, and color red was used for the adhesive layer. The component was characterized by a composite central core that was wrapped by an antitorsional layer. The lamination sequence of the composite rotor blade root cannot be disclosed for confidentiality reasons. Three passing-through holes were positioned in the curved part of the blade root: one of them was located in the symmetry centre, and the other two were located symmetrically to the central one. Consistent with the curved part, additional layers of composite plies were co-bonded over the antitorsional layer: this produced a tapering in the cross section, which is clearly visible in Figure 1a, colored light blue.

The antitorsional layer was connected to the central core by means of an adhesive layer, which is depicted in Figure 1a, colored red, and represents the focus of this work, since the SHMS was aimed at monitoring its structural integrity.

The following section describes the FE model of the blade root.

### 2.2. Finite Element Model

**Figure 2:**
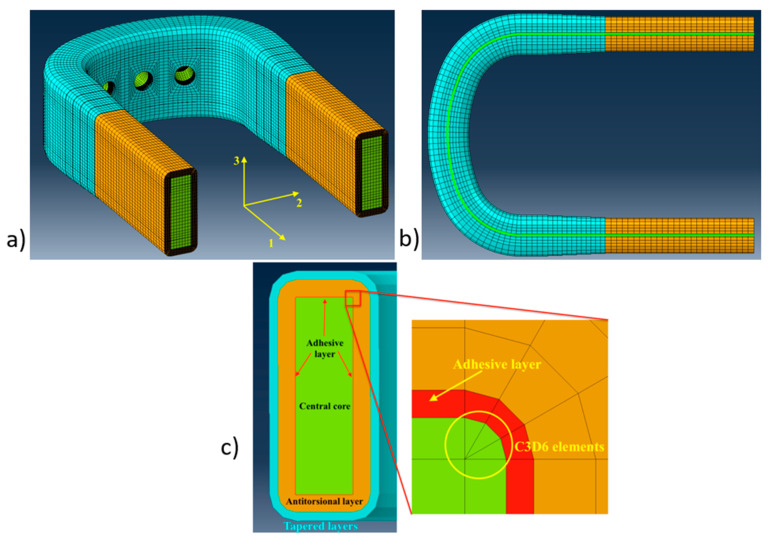
Meshing strategy adopted for the component: (**a**) the Finite Element model of the blade root; (**b**) the curvilinear path of the central core; and (**c**) the cross section with detail of the mesh of the corner region with C3D6-type elements.

The component was modeled with the software Simulia/Abaqus 2019, and the input file was automatically generated through a Matlab^®^ R2022b script. In Figure 2, the mesh adopted for the component is shown, evidencing the three types of elements that were adopted: hexahedral bricks, wedge elements, and cohesive elements (respectively, types C3D8, C3D6, and COH3D8 in the solver code [43]). The antitorsional layer and the tapered antitorsional layer were modeled ply by ply, with C3D8-type elements to correctly capture the state of stress. The central core was modeled with both C3D8- and C3D6-type elements.

As can be seen in Figure 2c, the C3D6-type elements were used only in the corners of the central core in order to allow the proper meshing of the fillet region, while in all the other locations, C3D8-type elements were adopted. The adhesive layer was modeled with the COH3D8-type cohesive elements having a thickness of 0.1 mm. Their penalty stiffness was set equal to the value calculated according to [44], considering, as the adherent thickness was *t*, the thickness of the antitorsional layer. Equation (1) related the penalty stiffness of the cohesive element to the out-of-plane stiffness and thickness of the adjacent layers:

(1) $$K=\alpha \frac{E_{33}}{t}$$

where *K* represented the normal penalty stiffness of the cohesive element and *α* represented a constant which was set equal to 50, according to [44]. *E*_33_ represented the normal out-of-plane modulus of the antitorsional layer. A total of 16 elements were used to mesh the antitorsional layer through the thickness, while for the central core, 7 elements were used along the cross section’s width, and 24 elements were used in the height of the cross section. For the antitorsional tapered layer, a total of 5 elements were adopted through its thickness. A characteristic element length of 3 mm along the curvilinear path was adopted.

**Figure 3:**
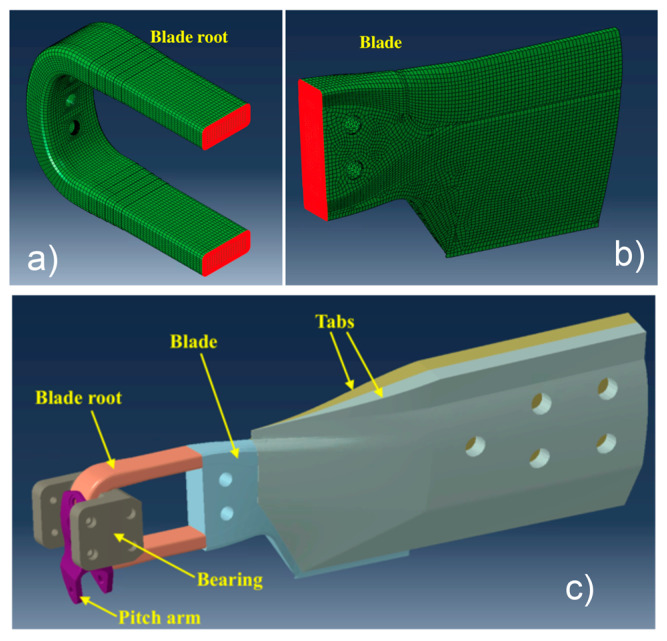
Blade root assembly: (**a**) blade root surfaces involved in the tie connection with the rest of the blade; (**b**) surfaces of the rest of the blade involved in the tie connection with the blade root; and (**c**) blade root assembly for testing.

The model of the component was introduced into a larger model representing the blade when subjected to a fatigue test that was actually performed on a test rig; all the components are shown in Figure 3 and are listed below:

- Blade root: Representing the component to be monitored. Its meshing strategy was presented in the previous subsection;
- Blade: Representing the part of the blade from the end of the blade root up to approximately half of the whole blade. The blade was modeled using C3D8I-type elements having a characteristic length of 5 mm;
- Tabs: These elements were bonded to the blade and were needed to transfer the load to the blade root. Tabs were modeled with C3D10-type elements having a characteristic length of 10 mm;
- Bearing: A metallic element, which was representative of the connection of the blade to the hub; the connection was implemented by three bolts passing through the holes of the blade root. The bearing was modeled with C3D8R-type elements having a characteristic length of 2.5 mm;
- Pitch arm: A metallic element that was bonded to the blade root and allows the introduction of loads to rotate the blade around a pitch axis. The pitch arm was modeled with C3D10-type elements having a characteristic length equal to 5.5 mm.

A nonlinear static FE model was developed, in which the blade root was connected to the rest of the blade with a tie interaction; the surfaces involved in such interactions are shown in Figure 3a,b, highlighted in red color. Tabs were also connected to the blade with a tie interaction, as well as to the pitch arm and to the blade root. A contact interaction between the blade root and the bearing was modeled, introducing a friction coefficient equal to 0.1.

### 2.3. Loading Scenario

**Figure 4:**
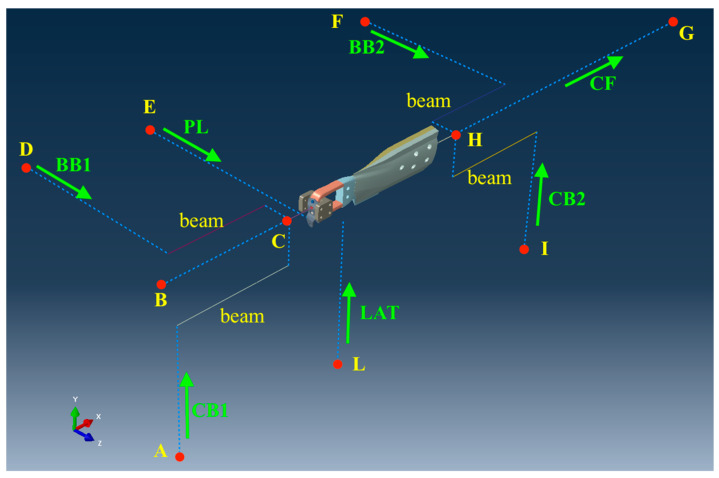
The constraints and load set applied to the blade during the testing in the test rig.

The loads applied throughout this work were defined considering the load set that is actually used to perform the fatigue testing for the blade certification, which in turn are chosen to represent realistic load conditions for the rotorcraft blade. In the experiments, the load set consisted of seven components that were applied through a proper test rig. In the FE model, such loads were applied by using connector elements (type CONN3D2 [43]). A description of the component of the load sets, as shown in Figure 4, is hereby provided:

- CF: centrifugal force, which was applied to the tabs;
- PL: force acting on the pitch link, which counterbalances the pitching moment;
- LAT: lateral load, which was applied by the lag damper connected to the blade;
- CB1 and CB2: in-plane bending loads, introduced through the bearing and through the tabs, respectively;
- BB1 and BB2: out-of-plane bending loads, introduced through the bearing and through the tabs, respectively.

Table 1 describes the constraints applied to the structure, most of them consisting of spherical hinges.

It can be observed that all the loads were concentrated, and they were applied statically to the model.

The analysis was performed in two steps: in step 1, a preloading of the bolts was performed so as to establish the contact between the blade root and the bearing, while in step 2, the external loads were applied to the blade. The load set applied, both in the experiment and in the model, was representative of the forces applied to the blade during its operational life.

### 2.4. Model Validation

The model was validated by performing an experiment, in which the blade was loaded statically in a test rig with all the components of the load set shown in Figure 4. The normalized value of each component is reported in Table 2. The load set was normalized with respect to the value of BB1 for confidentiality reasons. Strains were sampled by the strain gauges applied on the straight arms of the blade root, which are visible in Figure 1b.

The results of the validation of the nonlinear implicit model are presented in the second column of Table 3, and they were normalized with respect to the strain gauge number S7 for confidentiality reasons. The strain values were extracted from the FE model in the centroid position of the elements located on the surface, corresponding to the position of the strain gauges in the experimental set-up.

Considering the complexity of the component, the possible manufacturing defects, geometrical imperfections, and alignment imperfections in the tests, the pointwise correlation between the numerical and the experimental strains can be evaluated as acceptable.

However, the scope of the model involves the accomplishment of a large number of analyses in order to train and test the ANN that was used for the damage and load detection. For this reason, it was deemed of primary importance to reduce the computational cost. Consequently, the model was adapted to perform linear analyses. A linear model was created, starting from the nonlinear one and removing the contact interactions. In particular, the contact between the blade root and the bearing was removed and replaced with a tie interaction. However, it was observed that the relative tangential motion that occurred in the presence of the contact helped in reducing the shear stresses transmitted from the blade to the bearing. A better representation of the real working condition was accomplished by using a layer of cohesive elements (COH3D8 [43]) with a very low shear penalty stiffness (70 N/mm^3^) so as to reduce the transmitted shear stresses, as occurs in the case of a contact interaction. The cohesive elements were interposed between the bearing and the blade. The normal penalty stiffness was set as equal to the value adopted for the adhesive interface.

The results of the linear model validation are shown in third column of Table 3. As it can be observed, a non-negligible mismatch was identified between the linear model and the nonlinear one. The reason for such a difference lies in the contact nonlinearity occurring between the blade root and the bearing. Although this phenomenon was partially mitigated by using a layer of cohesive elements with a low penalty stiffness in the linear model to release the shear stresses transmitted through the contact, the behavior of the contact in the out-of-plane direction could not be replicated. The percentage errors with respect to the experiments are higher for the linear model. However, since the scope of this paper was an evaluation of the performances of an SHMS applied to a real-world thick composite element with complex geometrical features and in combined load conditions, the linear model was considered an acceptable approximation of a real blade root. Indeed, for a real application, a more detailed model identification has to be performed to make available a high-fidelity model that is capable of correctly predicting the strain field evolution due to load variation and damage occurrence, so as to adopt it in the training phase of the ANN algorithm development. This can be achieved by exploiting the high number of FBG sensors embedded in the component during the manufacturing stage, which can be used to validate the FE model on a higher number of points, not only located on the surface, but also located internally. Once a high-fidelity model is obtained, modern high-performance computing can certainly provide the resources for the application of the method to a fully nonlinear model.

## 3. The Damage Detection Algorithm and Virtual SHMS Description

The damage detection procedure was characterized by three main steps [45]: (i) anomaly detection, whose task was to detect the presence of damage, (ii) damage assessment, whose task was to provide an estimation of the damage’s size, and (iii) damage localization, which provided the information about the location of the damage on the component.

The main problem related to this specific application was that the strain field was affected by the presence of damage and by the application of the load. In order to detect a possible presence of damage, these two aspects were decoupled. Three versions of the algorithm were evaluated and compared, considering their noise sensitivity and damage detection performance. In this study, the SHMS was represented by virtual sensors, represented by elements of the FE model, and the strain was obtained by sampling the strain values from the element located on the optical fiber path. For this study, the machine learning toolbox of Matlab^®^ R2022b was adopted.

### 3.1. Virtual SHMS for Strain Acquisition

As it was presented in the introduction section, this work was based on a previous study [1], which investigated the technological feasibility of an SHMS based on FBG sensors in a composite helicopter rotor blade. In that work, the authors adopted a customized interrogation system, which was designed to withstand high accelerations because it was mounted on the rotor hub to read the signal of 64 FBG sensors. In this work, to assess the performance of the SHMS, a virtual sensing system was considered, embedded in the blade root. Some of the elements of the FE model were supposed representing the FBG sensors: one element was treated as one FBG sensor, since the characteristic length of the elements, about 3 mm, was comparable to the possible length of a real FBG sensor. By taking the strains at the centroid of the element, the obtained strain could be considered a measure of the average strain in the FBG when located in the correspondent position. The strain values were obtained by rotating the entire strain tensor of 45° and considering only the strain component that was directed along the optical fiber path ε_11_ of the rotated tensor. Certainly, the assumption behind this procedure is that the strain read by the sensor corresponds to the strain of the host material; actually, the protective layer of the optical fiber can affect the measure of the strain of the sensor [46,47]. This aspect was addressed in [1], where the authors adopted a coating made of ORMOCER to guarantee a satisfactory adhesion to the host material; therefore, in this work, a perfect strain transfer was assumed.

**Figure 5:**
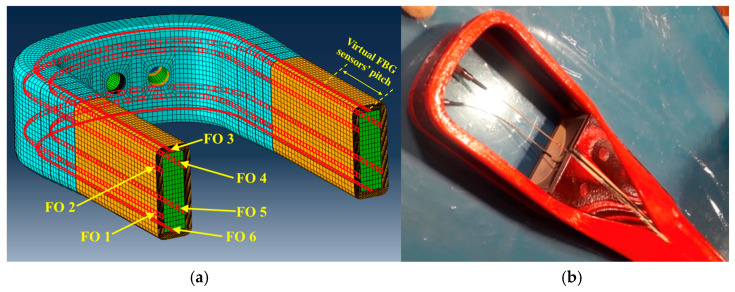
Virtual and real optical fiber-based sensors in a composite blade: (**a**) the virtual optical fiber paths chosen in this work; (**b**) an example of optical fibers integrated in a helicopter tail rotor blade, adapted from [1] with permission.

To be consistent with the technological aspects regarding the embedment of the FBG sensors in a composite laminate, the virtual FBG sensors were supposed as belonging to six virtual optical lines, with the paths shown in Figure 5a, colored red, while in Figure 5b an example of optical fiber sensors integrated in a composite helicopter tail rotor blade can be observed. The virtual optical fiber paths were considered located inside the antitorsional layer, colored orange, at a distance of 0.25 mm from the adhesive layer.

A number of 10 virtual FBG sensors per optical fiber was considered, consistent with the limitations provided by the multiplexing technique [48], so that a total of 60 virtual FBG’s were employed in the SHMS. The virtual sensors were distributed equally spaced along each optical fiber with a pitch equal to 42 mm, which is highlighted in Figure 5a.

### 3.2. Damage Detection Algorithms

The basic strategy for damage detection was based on decoupling the effects of the load application from the effects of the damage by first identifying the applied load set and subsequently identifying the possible presence of damage. The assumption behind this strategy was that the load set could be recognized with a satisfactory accuracy despite the perturbation that the damage created to the strain field. This assumption was verified in the next section.

The three versions of the algorithm differed only in the first stage, which was the load identification step. In version #1, the load identification was performed with the ANN trained only on FE analyses of the blade in pristine configurations, while in version #2, the load identification was performed with the ANN trained on FE analyses of the blade in pristine and damaged configuration. In both version #1 and version #2, the identification of the load was performed using an ANN for regression. The architecture of this ANN, hereafter named as ANN 1, was characterized by three layers, each one composed of 10 neurons. Of the dataset, 70% was used for the training, 15% for validation, and the remaining 15% for testing. More details about the architecture and the training parameters are reported in Table 4.

The ANN 1 took in input of the strain values of the sensors and provided an output of the identified load set. In version #3, the load identification was performed by finding the load set from the strain measures computing the pseudo-inverse of the calibration matrix. Actually, the solution to the problem of the load set is known to be particularly affected by the bad conditioning of the system [49]. The load identification, assuming a linear response of the model, was based on Equation (2), which related the vector of the applied load set to the strain values acquired at the sensor position:

(2) $$\left\{\begin{array}{c} \varepsilon_{1}\\ \vdots \\ \begin{array}{c} \varepsilon_{m} \end{array} \end{array}\right\}=\left[\begin{array}{ccc} K_{11} & \cdots & K_{1n}\\ \vdots & \ddots & \vdots \\ K_{m1} & \cdots & K_{mn} \end{array}\right]\left\{\begin{array}{c} P_{1}\\ \vdots \\ \begin{array}{c} P_{n} \end{array} \end{array}\right\}$$

where {*ε*} represented the vector containing the strain values of each sensor, [*K*] represented the calibration matrix obtained by the FE model, and {*P*} represented the load vector. The size of matrix [*K*] was *m* × *n*, where *m* represented the number of strain sensing points, and *n* represented the number of load components. Accordingly, the size of {*ε*} was *m* × 1, and the size of {*P*} was *n* × 1. Consistently with the assumption of the negligible effect of the damage on the overall strain field involved in load identification, the calibration matrix [*K*] was calculated on the blade in pristine condition. The load set was then identified by computing the pseudo-inverse of the calibration matrix, presented in Equation (3).

(3) $$\left\{P\right\}={\left({\left[K\right]}^{T}\left[K\right]\right)}^{-1}{\left[K\right]}^{T}\left\{\varepsilon \right\}$$

where {*P*} represented the load set to be identified, and [*K*]*^T^* represented the transposed values of the calibration matrix [*K*]. Table 5 shows a summary of the load identification methodologies for all the three versions of the algorithm.

Once the load set was identified, regardless of the algorithm’s version, Equation (2) was used to calculate the nominal strain values that would be provided by the sensors for the identified load condition with the blade in pristine condition. Such a process represented a simplification that was allowed by the linearity of the model. If the nonlinear model had to be considered, the architecture of the damage detection process would not change, but the strains would need to have been evaluated through a nonlinear analysis performed with the identified load set. By comparing the calculated strain on the notional model and the actual strain read by the sensors, it was possible to define a damage index for each sensor that was representative of the structural integrity of the blade; the proposed damage index was reported in Equation (4).

(4) $${damage\;index}_{i}={\left(\varepsilon_{i}^{calculated}-\varepsilon_{i}^{measured}\right)}^{2}$$

where *ε_i_^calculated^* represented the strain value of the *i*th sensor calculated using Equation (2), while *ε_i_^measured^* represented the strain value measured by the sensor. An ANN for pattern recognition was then adopted to detect the presence of damage based on the damage indexes previously calculated; the ANN took in input the vector of the damage indexes and provided a scalar output which was compared to a threshold value. The blade was considered damaged if the output exceeded a predefined threshold. If damage was detected, the next step of the algorithm was related to the identification of the damage’s dimension and its location [45]. To perform this task, an ANN for regression was adopted.

All the versions of the algorithm were trained, validated, and tested by adding Gaussian noise with a zero mean and standard deviation equal to 1%, 2%, 4%, or 6% of the nominal strain value provided by the sensor (in this case, from the strain sampled from the FE model). For all the versions of the algorithm, the anomaly detection was performed with an ANN for pattern recognition, hereafter named ANN 2, whose architecture is described in Table 4. The damage assessment and localization were performed with an ANN for regression, hereafter named ANN 3, whose architecture is described in Table 4. ANN 3 took in input of the damage index pattern and provided an output of the damage’s size and location. Regarding ANN 2 and ANN 3*,* 70% of the dataset was used for the training, 15% for validation, and the remaining 15% for testing.

**Figure 6:**
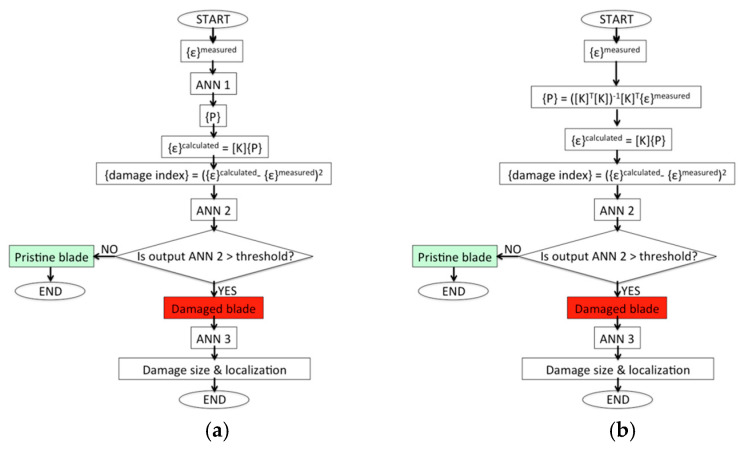
Flow diagrams of the damage detection algorithms: (**a**) Damage detection algorithm diagram for version #1 and version #2; (**b**) damage detection algorithm diagram for version #3.

The architecture for each ANN was chosen based on preliminary studies aimed at identifying the configuration providing better quality results; this operation led us to use three hidden layers for ANN 1 and ANN 2 and two hidden layers for ANN 3. The flow diagrams of the damage identification algorithm are presented in Figure 6 for each of the algorithm’s versions. In this case, Figure 6a refers to both version #1 and version #2, since the only difference between them was related to the condition of the blade on which the ANN 1 was trained.

### 3.3. Virtual Dataset Creation and Training

The most important interfaces in the blade root were the ones represented by the adhesive joints. These were critical because they were obtained through secondary bonding or co-bonding processes, involving parts that had been already cured. It was exactly for this reason that attention was focused on such interfaces, simulating damages that could be originated during manufacturing, which can propagate during the operational life of the helicopter.

**Figure 7:**
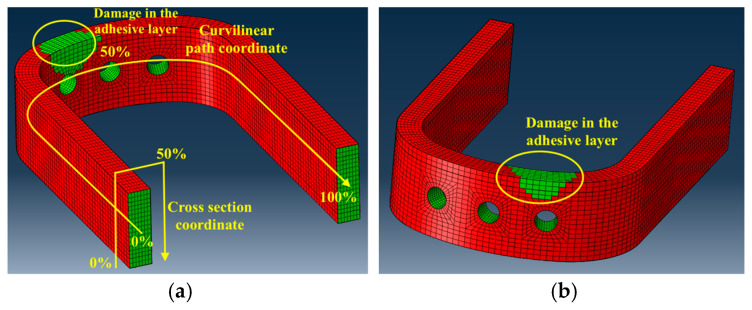
Damage introduction in the FE model; (**a**) the coordinate system for damage introduction and an example of damage of diameter 30 mm, located at 44.44% of the curvilinear path, and 50% of the cross section coordinate, better shown in (**b**).

To create the virtual datasets to train and test the ANNs, different FE analyses were performed, introducing damages during the generation of the analysis’ input file that deleted the cohesive elements that were included in a space described as a sphere that was located on the surface of the adhesive layer to be monitored. Such damages were introduced in 40 different locations on the adhesive: in detail, referring to Figure 7a, damages were introduced in four locations equally spaced along the cross section coordinate and 10 locations equally spaced along the curvilinear path. Only one damage per FE analysis was created in the adhesive. It must be specified that, for each combination of a curvilinear path coordinate and a cross section coordinate, nine damage dimensions were analyzed, having the following sizes: 3, 6, 9, 12, 15, 18, 21, 24, and 30 mm. A total of 360 damage scenarios were considered in this study, resulting in the combination of 10 damage locations in the curvilinear path coordinate, 4 damage locations in the cross section coordinate, and 9 damage dimensions. Figure 7a,b shows an example of damage of 30 mm diameter, located at 44.44% of the curvilinear path, and at 50% of the cross section coordinate.

To create a dataset considering different load conditions and to limit the number of FE analyses to be performed, the linearity of the FE model was exploited. A calibration matrix was calculated for each damage size and position, performing an FE analysis by applying one unitary load per time. Once the calibration matrices were obtained (each one specific for damage size and position), different strain fields were obtained by multiplying the calibration matrix by the load condition, representative of the operational loads. Two load sets were considered to create the training dataset, hereafter named as load set A and load set B, each one varying according to the following laws, represented in Equation (5) and Equation (6) for load set A and load set B, respectively. The form of these equations follows the variation of loads used in the fatigue testing of the blade, mentioned in Section 2.3, which in turn were defined considering the real load conditions experienced by the blade.

(5) CF=122.27BB1=0.67+0.33sin⁡θCB1=6.1+1.4 sin⁡θLAT=8.89+3.33sin⁡θPL=2.22+1.11sin⁡θBB2=0.67+0.33sin⁡θCB2=−6.11−1.55sin⁡θ

(6) $$\left\{\begin{array}{c} CF=106.24+106.24\sin\theta \\ BB1=0.88+0.88\sin\theta \\ \begin{array}{c} CB1=6.66+6.66\sin\theta \\ LAT=8.89+8.89\sin\theta \\ \begin{array}{c} PL=2+2\sin\theta \\ \begin{array}{c} BB2=0.88+0.88\sin\theta \\ CB2=-7.22-7.22\sin\theta \end{array} \end{array} \end{array} \end{array}\right.$$

To generate realistic load conditions for the ANN training, the parameter θ was varied in the range 0 ÷ 2π, thus generating 100 evenly spaced load conditions, varying CF, BB1, CB1, LAT, PL, BB2, and CB2, which represent the load components presented in Figure 4.

It is worth remarking that also in the former equations, loads were normalized with respect to the maximum peak value of BB1 of load set A for confidentiality reasons. The normalization is the same as the one adopted for the loads presented in Table 2, referred to as the experimental load scenario. By applying Equations (5) and (6), a total of 200 load conditions were generated. It must me remarked that to generate the training dataset, the load components were applied together to the model, such as to obtain a realistic load condition in which the blade was subjected to the superposition of different type of loads, which were chosen to represent an approximation of the operational conditions as explained at the beginning of Section 2.3.

For the stage of load identification, in version #1, the training of ANN 1 was performed on 70% of a dataset made of 8000 load conditions and was obtained by repeating 40 times the 200 load conditions and adding Gaussian noise. Considering the dataset for the ANN 1 of version #2, the same number of load conditions for pristine and damaged conditions were considered, each one composed of 8000 load conditions. The input of ANN 1 was represented by the strain acquired at the sensor location and the output was represented by the identified load set.

Considering the dataset for the ANN 2, the latter was equally divided in two subsets; one of them was characterized by only pristine conditions and the other one was characterized by only damaged conditions, obtaining a balanced dataset, as suggested in [38]. Each of the 200 load conditions was combined with the 360 damage scenarios, obtaining a dataset made of 72,000 damage index patterns. The same number was then produced with the blade in pristine condition by repeating each load condition 360 times, adding Gaussian noise, and calculating the related damage index patterns. The input of ANN 2 was represented by the damage indexes calculated for each sensor, according to Equation (4), and the output was represented by a scalar value between 0 and 1. The blade was then classified as pristine or damaged, depending on the chosen threshold value.

Considering ANN 3, the related dataset was made only of damaged conditions, therefore, only the part of the dataset of ANN 2 related to the damaged conditions was used to train and test ANN 3: this dataset was then divided into training/validation/testing, according to the partition presented in Table 4. The input of ANN 3 was characterized by the damage index pattern, as it was for ANN 2, and the output was represented by the estimated position and the damage size.

In this case, the baseline was represented by a pristine blade root, without any defect in the adhesive layer; however, in some cases, the presence of manufacturing-induced defects can modify the baseline. The presented approach can also be applied in this scenario, provided that the analyzed “damaged cases” are consistent with the possible evolution of the initial defect.

## 4. Results

In this section, the results of all the algorithm versions are presented in terms of load identification, anomaly detection, damage assessment, and localization. The performances of all the versions of the algorithm were assessed for different noise levels, namely 1%, 2%, 4%, and 6%. Since the dataset was divided randomly for training, validation, and testing, in order to obtain a more reliable estimation of the performance of the algorithm, the results were averaged for 10 algorithm runs.

### 4.1. Example of Damage Identification

**Figure 8:**
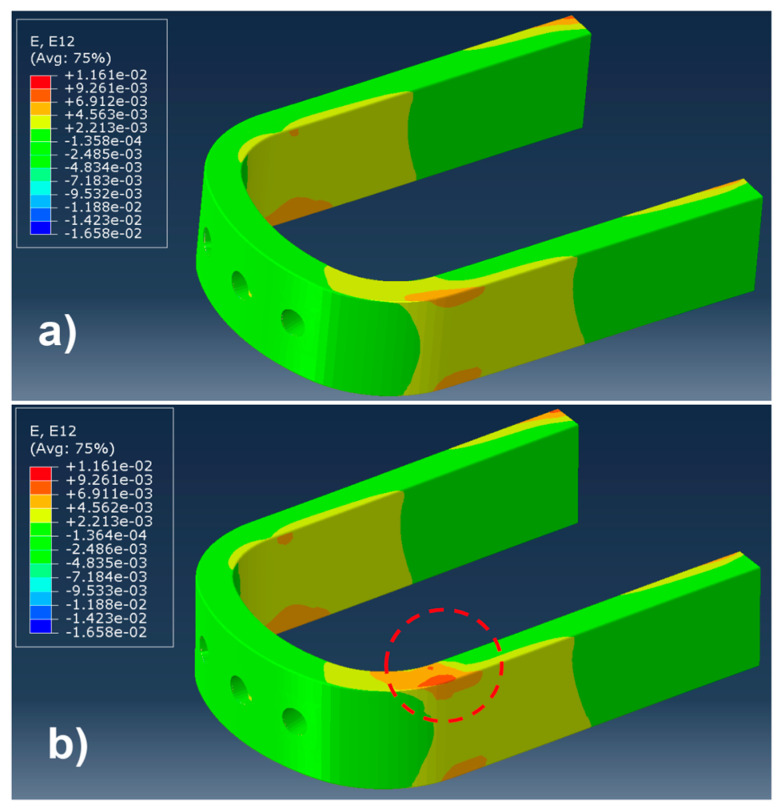
An example of a strain field in the blade root: (**a**) strain γ_12_ field in a pristine blade root; (**b**) strain γ_12_ field in a damaged blade root. Both of them were subjected to the same load condition, presented in Table 6.

In this subsection, an example of damage identification was provided to better clarify the process through which damage was identified in the blade root. The example hereby considered consisted of two different blade roots, one without damages and the other one having damage of 30 mm size, located at a 33.33% of the curvilinear coordinate, and at 49.95% of the cross section coordinate, according to the coordinate systems presented in Figure 7a. The same load set was applied to both the damaged and undamaged blade roots, whose values were reported in the first row of Table 6, normalized with respect to the load component BB1 for confidentiality reasons. In Figure 8, the strain field contour can be observed for the case of a pristine blade root, presented in Figure 8a, and a damaged blade root, presented in Figure 8b. A difference in the contour can be observed in the red circle, where the damage was introduced, in Figure 8b.

In this example, version #1 of the algorithm was considered with 2% of Gaussian noise on the strain data. The identified load set, recognized using ANN 1, can be observed in the second row of Table 6 for the pristine blade root, while in the third row, the load set recognized in the damaged blade root can be observed. It can be seen that the load set was slightly better recognized in the pristine blade root. The input of ANN 1 was represented by the strain field obtained by the 60 virtual FBG sensors, and the output was characterized by the seven load components of the load set.

According to the procedure previously described, and depicted in Figure 6a, the anomaly detection step was accomplished. Therefore, a damage index pattern was calculated for each virtual FBG sensor on the basis of the identified load set using Equation (4). The calculated damage index pattern was then given as the input to ANN 2, whose task was to detect the presence of anomalies. The output of ANN 2 was represented by a scalar value between 0 and 1, where 0 represented the target for the pristine blade root, while 1 represented the target for the damaged blade root. In this example, the following outputs of ANN 2 can be observed for the pristine and damaged blade root:

- Output of ANN 2 for the pristine blade root = 0.26;
- Output of ANN 2 for the damaged blade root = 0.99.

In this example, a threshold equal to 0.5 was chosen, above which a blade root was considered as damaged, while a value below 0.5 was considered pristine. According to this choice, the damaged blade root was correctly classified as damaged, and the pristine blade root was correctly classified as pristine.

Once the anomaly detection step was accomplished, the next step consisted of the damage assessment and localization, which were performed using ANN 3. According to the Rytter’s hierarchy [45] this step needs to be accomplished only if a structure was classified as damaged in the step of anomaly detection; therefore, only the damaged blade root was considered in this example. The size and position of the damage were estimated by ANN 3, whose input was represented by the damage index pattern, and the output was represented by the size, the curvilinear coordinate, and the cross section coordinate of the damage. The results were reported in Table 7, where in the first row the actual values of damage size and position were presented, while in the second row, the size and position of the damage as estimated by the algorithm were presented.

An example of damage identification was provided in this subsection, which aimed to better understand the results obtained in the next subsections, in which the performances of the different versions of the algorithm were assessed and compared on a wider range of load sets, damage sizes, and positions.

### 4.2. Load Identification

**Figure 9:**
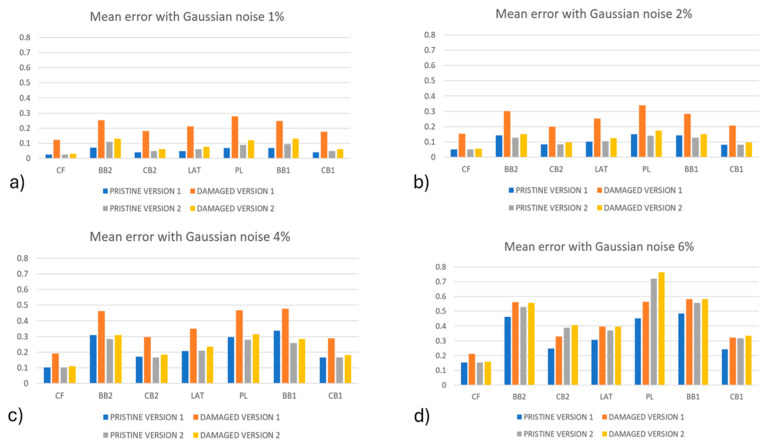
The mean percentage error of load identification for algorithm version #1 and version #2 for (**a**) 1% Gaussian noise, (**b**) 2% Gaussian noise, (**c**) 4% Gaussian noise, and (**d**) 6% Gaussian noise.

The results regarding load identification for algorithm version #1 and version #2 are provided in Figure 9, in which the average error was calculated for each load component. Moreover, a comparison between the identified loads on the pristine and damaged blades is shown. The error was normalized with respect to the maximum magnitude of each load component.

As expected, the error for all the load components increased as the noise level increased. For example, an increase in the maximum mean error of 0.27% for the PL load component with 1% noise level to a maximum average error of 0.76% for the same load component with a noise level of 6% was identified. It can be observed that in all the cases, the error related to the damaged conditions was higher than the one obtained for the pristine condition, but this difference decreased when the noise level increased This is clearly observed in Figure 9d, where the noise level reached 6%: a possible explanation for this behavior is related to the fact that damage perturbs the strain field locally, with an effect that is comparable to the effect of the noise. So, as the noise level was increased, it tended to cover the effect of the damage. In the comparison between the algorithm’s versions, it can be observed that version #1 of the algorithm provided a higher difference between the errors in the pristine and damaged conditions than version #2; however, these differences decreased by increasing the noise level.

**Figure 10:**
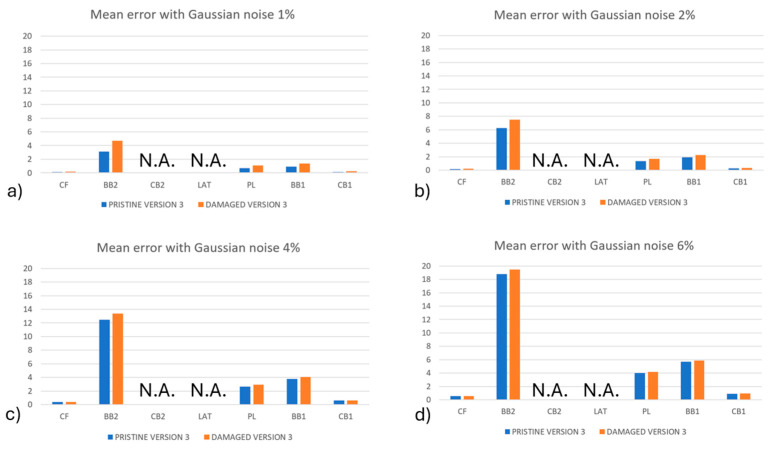
The mean percentage error of load identification for algorithm version #3 for (**a**) 1% Gaussian noise, (**b**) 2% Gaussian noise, (**c**) 4% Gaussian noise, and (**d**) 6% Gaussian noise. Load components CB2 and LAT were not reported because they were off-scale, accordingly, the notation N.A. was added to the figures.

In Figure 10, the mean percentage error of load set identification for version #3 of the algorithm is shown. As already mentioned, the loads were obtained by solving for the load set {*P*}, as in Equation (3). Errors related to the load components CB2 and LAT were not reported because they were off-scale for the plot: the error was 5 to 20 times the maximum magnitude of the loads. Regardless, their contributions to the strain fields were verified to be one order of magnitude lower than the other loads that were acting on the blade; this may be the reason for such high errors.

The same behavior for version #1 and version #2 of the algorithm was observed. By increasing the noise level, the error increased as well; moreover, for all the noise levels and for all the load components, the damaged configuration provided higher errors. It was interesting to observe that, also in this case, by increasing the level of noise, the difference between the errors in the pristine and damaged conditions were reduced. However, the load identification method of version #3 provided worse results than version #1 and version #2 of the algorithm: the load set identification by means of the ANN 1 revealed itself to be much more effective with respect to solving the inverse problem for the load set {*P*}, as in Equation (3).

### 4.3. Anomaly Detection

The effectiveness of the algorithm to detect the possible presence of damage was evaluated considering the Receiver Operating Characteristic (ROC) curves, which allowed for the expression of the Probability Of Detection (POD) function of the Probability of False Alarm (PFA), varying the threshold over which a structure was considered as damaged. Such a criterion was chosen as it allows for evaluating how much the algorithm is able to distinguish a damaged blade from a pristine blade. In fact, this is a key aspect in the cost effectiveness of SHMSs, which have to maximize the POD while minimizing the PFA. The ROC curves were derived for all the noise levels (1%, 2%, 4%, and 6%) for each damage dimension and considered all the three versions of the algorithm. The preliminary results suggested that the algorithm was not sensitive to small damages, as the related ROC curves were positioned on the no-performance line: the diagonal of the ROC plot that represents a random classifier. To improve the detection performances for high damage dimensions, since no performance was obtained with lower ones, the ANN 2 was trained not considering the damages with smaller sizes, like 3 mm, 6 mm, and 9 mm, as suggested in [38], maintaining the same dataset partition between pristine and damaged cases. The POD and PFA were calculated according to the “HIT/MISS data” method presented in [50]. The POD was calculated considering damaged conditions and counting the number of times that the output of the ANN 2 exceeded the threshold value. This was then divided by the total number of damaged conditions and analyzed by the ANN 2, as reported in Equation (7).

(7) $$POD=\frac{TP}{TP+FN}$$

where *TP* represented the True Positives (damaged conditions classified as damaged) and *FN* represented the False Negatives (damaged conditions classified as pristine); their sum gave the total number of damaged conditions analyzed by the ANN 2.

The PFA was calculated considering instead pristine conditions, the number of times that the output of the ANN 2 exceeded the threshold value was divided by the total number of pristine conditions analyzed by the ANN 2, as reported in Equation (8).

(8) $$PFA=\frac{FP}{FP+TN}$$

where *TN* represented the true negatives (pristine conditions classified as pristine) and *FP* represented the false positives (pristine conditions classified as damaged), their sum represented the total number of pristine configurations analyzed by the ANN 2.

The ROC curves were then obtained by calculating the POD and the PFA, varying the threshold value from its minimum to its maximum value: in this specific case, between 0 and 1.

**Figure 11:**
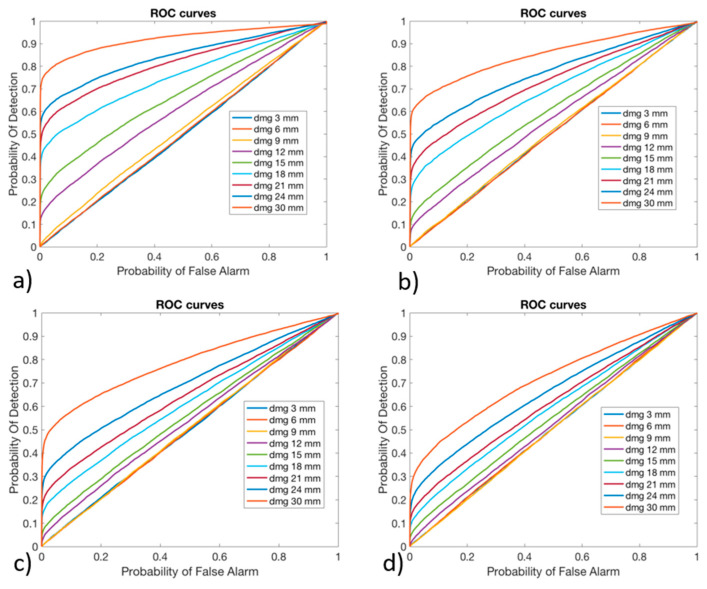
Receiver Operating Characteristic curves for algorithm version #1, obtained with (**a**) 1% Gaussian noise, (**b**) 2% Gaussian noise, (**c**) 4% Gaussian noise, and (**d**) 6% Gaussian noise.

**Figure 12:**
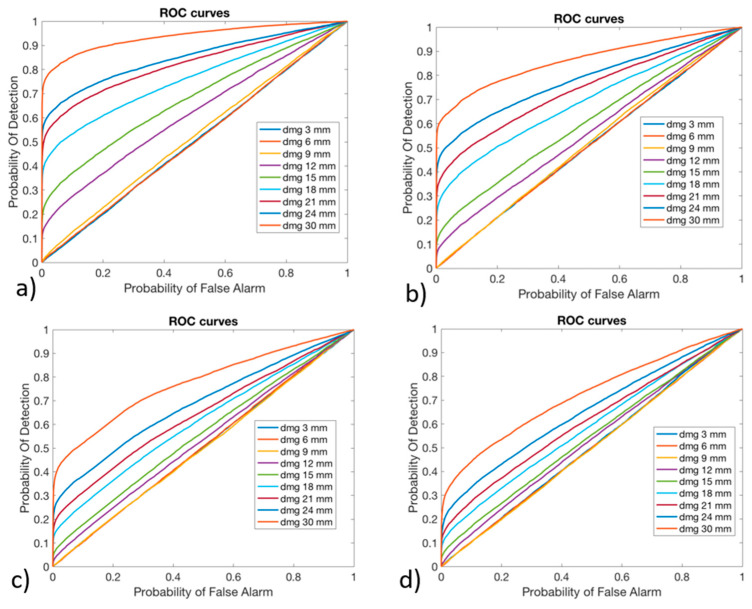
Receiver Operating Characteristic curves for algorithm version #2, obtained with (**a**) 1% Gaussian noise, (**b**) 2% Gaussian noise, (**c**) 4% Gaussian noise, and (**d**) 6% Gaussian noise.

**Figure 13:**
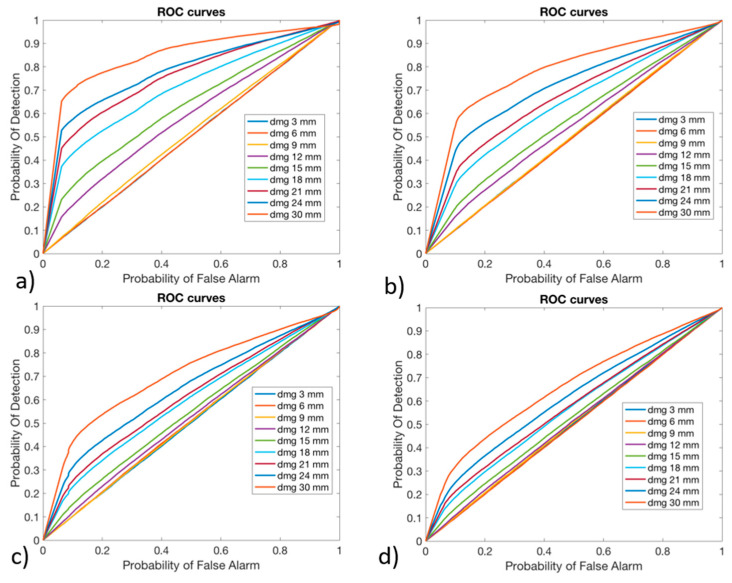
Receiver Operating Characteristic curves for algorithm version #3, obtained with (**a**) 1% Gaussian noise, (**b**) 2% Gaussian noise, (**c**) 4% Gaussian noise, and (**d**) 6% Gaussian noise.

In Figure 11, Figure 12 and Figure 13, the ROC curves for version #1, version #2, and version #3 of the algorithm, respectively, are shown.

As can be observed, the damages with a higher dimension were more likely to be detected: their curve is in the upper-left side of the plot. The damages with lower dimensions, like 3 mm, 6 mm, and 9 mm, could not be identified by any one of the versions of the algorithm: their curve is on the no-performance line corresponding to the segment connecting the axis origin to the point (1,1). As was expected, by increasing the level of noise, the algorithm decreases its performance, reducing the POD for the same PFA. Version #1 and version #2 of the algorithm seemed to provide comparable results in the anomaly detection, while version #3 of the algorithm provided lower performance.

In fact, especially for small values of PFA (where the optimal threshold should be chosen), the POD showed very small values compared to version #1 and version #2 of the algorithm.

### 4.4. Damage Assessment and Localization

Once the presence of damage was detected, the subsequent step was related to its assessment and localization. Indeed, these two steps provided information that was useful for decision making. For instance, depending on the estimated damage size, a fast decision can be made about repairing or substituting the blade, without the need to inspect the blade with NDT. The localization of the damage allows an automatic identification of its position, making possible the evaluation of the effects of the damage in that specific point of the structure.

**Figure 14:**
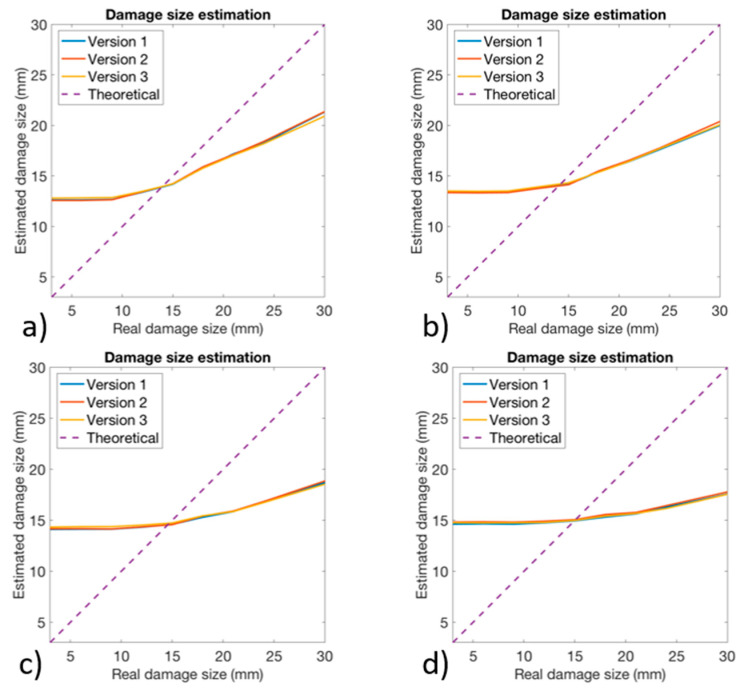
Damage quantification obtained for all the three versions of the algorithm with (**a**) 1% Gaussian noise, (**b**) 2% Gaussian noise, (**c**) 4% Gaussian noise, and (**d**) 6% Gaussian noise.

The ANN 3 was trained only on damaged configurations: damage size and position were given together to the ANN 3 as preliminary studies revealed that no improvement would be obtained by using two different ANNs for damage assessment and localization. In Figure 14, the plot of the real damage size and the estimated damage size is shown for all of three versions of the algorithm and for each level of noise. The dashed line colored in purple represented the theoretical behavior that the algorithm should have. As its angular coefficient is one, it means that for each real damage size the algorithm should provide exactly that one. The higher is the performance of the algorithm, the more its behavior is close to the theoretical one.

For damages with a reduced size, it can be seen that the algorithm overestimated the damage size, maintaining an estimation of about 15 mm; such behavior was observed for real damage that was sized below 15 mm. As the real damage size increased above 15 mm, the algorithm seemed to provide a more accurate estimation of the size of the damage. Regardless, the behavior was still far from the theoretical behavior. No significant differences existed between the three versions of the algorithm; it seemed that version #2 provided slightly better results than the others; however, this difference seemed to be very low. The main factor affecting the performance of the algorithm was represented by the noise level: as the noise level increased, the algorithm seemed to become less sensitive to the size of the damage. In particular, it was observed that the slope of the curves decreased as the noise level increased. Moreover, it seemed that the noise affected all the versions of the algorithm in the same way. The low sensitivity to the damage size may be due to the relatively low effect that damage in the adhesive produces. Indeed, the central core was much stiffer than the antitorsional layer. Therefore, a disbonding has little influence on the strain field and, consequently, the damage size estimation resulted to be difficult. Moreover, the presence of other structural elements like the pitch arm and the bearing contributed locally to increase the stiffness of the blade root, making the estimation of the damage size even more difficult.

**Figure 15:**
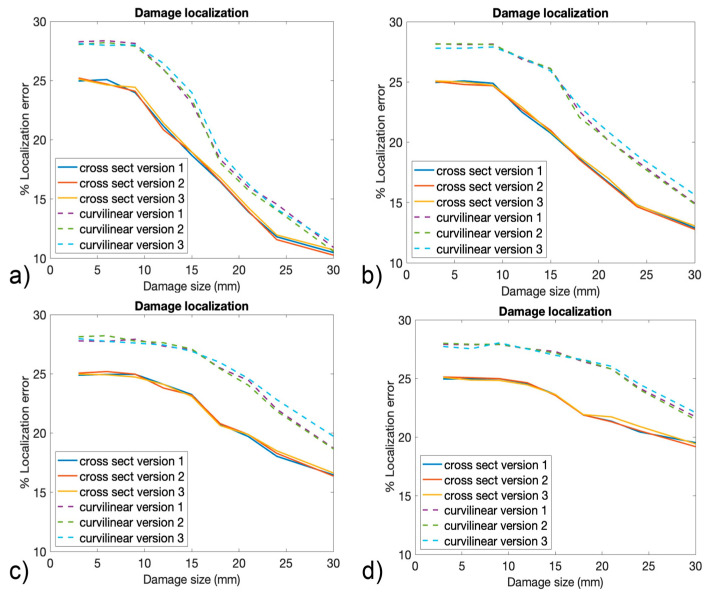
The percentage error in damage localization obtained for all three versions of the algorithm with (**a**) 1% Gaussian noise, (**b**) 2% Gaussian noise, (**c**) 4% Gaussian noise, and (**d**) 6% Gaussian noise.

The performances of damage localization are presented in Figure 15, expressed in terms of the mean percentage errors related to the cross section and curvilinear coordinates that were represented in Figure 7a. The percentage error was calculated considering, for each of the two coordinates, the absolute value of the difference between the real damage coordinate and the damage coordinate estimated by the ANN 3. The coordinates were expressed in percentage of the total length.

It was interesting to observe that for small damage dimensions, the localization error for both the coordinates was slightly affected by the level of noise, and it maintained a value of 28% and 25% for the curvilinear and cross section coordinates, respectively. Moreover, for small damage sizes, the performances of all the versions of the algorithm seemed to be similar. As the dimension of the damage increased, the error decreased for both the coordinates, and the algorithm seemed to provide lower performances as the noise level increased. In fact, it can be observed that for a damage size of 30 mm, the error for the cross section coordinate passed from 10–11% with a noise level of 1% to an error of 19% with a noise level of 6%. In general, no significant differences existed between the three versions of the algorithm, especially for low levels of noise. Overall, version #3 seemed to provide slightly higher errors for the curvilinear coordinates for higher levels of noise, while version #1 and version #2 exhibited similar behavior. The oscillation of the blue dashed line, corresponding to the curvilinear coordinate and version #3 of the algorithm observed in Figure 15d, can be explained considering the low sensitivity of the algorithm to small damage sizes. For instance, in the region from 3 mm to 12–15 mm, the Gaussian noise affecting the data played a more relevant role compared to higher damage sizes, thus leading to oscillations in a region where, for low noise levels, the curve was flat.

**Figure 16:**
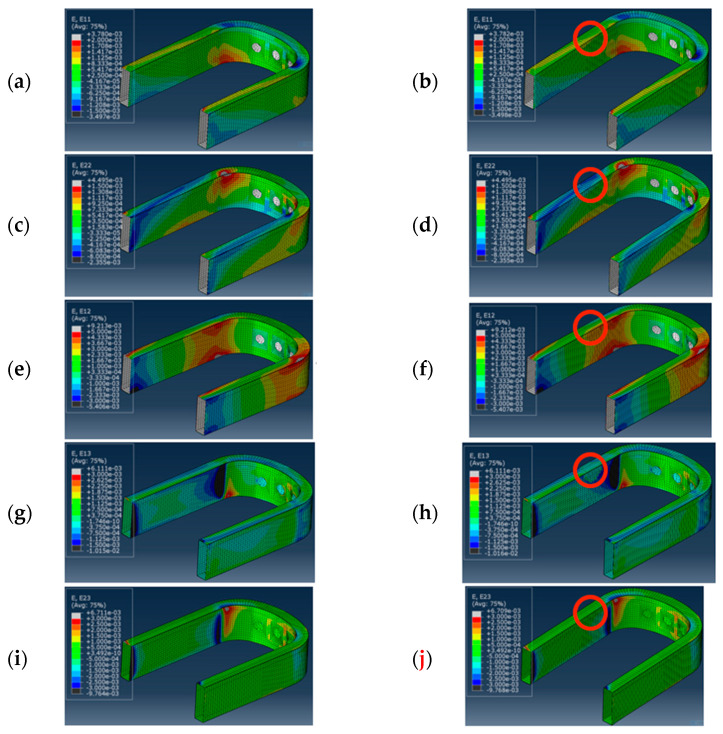
Strain field distribution in the elements’ local reference system of pristine blade root and damaged blade root, with a damage of 30 mm diameter, located at 50% of cross section coordinate, and 22.22% of curvilinear path coordinate, corresponding to the red circle: (**a**) ε_11_ pristine, (**b**) ε_11_ damaged, (**c**) ε_22_ pristine, (**d**) ε_22_ damaged, (**e**) γ_12_ pristine, (**f**) γ_12_ damaged, (**g**) γ_13_ pristine, (**h**) γ_13_ damaged, (**i**) γ_23_ pristine, and (**j**) γ_23_ damaged.

Figure 16 represents a comparison between the strain field distribution in a damaged and an undamaged blade root. The elements represented are related to the ply containing the virtual optical fibers. Considering the size of the damage, which was, in this case, equal to 30 mm, it should be observed that the strain distribution was almost not affected by the presence of the damage.

Only one strain component seemed to be influenced by the presence of the damage, which is represented in Figure 16h, and it was the out-of-plane shear γ_13_; however, such a strain component is particularly difficult to measure with an FBG sensor, as they are sensitive to the deformation along the optical fiber path.

## 5. Conclusions

The performance of an SHMS was evaluated on a composite rotor blade root that was subjected to a variable loading condition. The component was modeled with FE, and the elements located on the path of the virtual optical fibers were supposed by providing the strain read by the FBG sensors. The FE model was previously validated with experiments of loading the blade root in a test rig and applying seven different loads that were representative of the real operational conditions. Damages of different dimensions and in different positions were introduced in the adhesive layer between the central core and the antitorsional layer. An algorithm based on ANN was proposed to identify the presence of damages under variable loading conditions. Three different versions of the algorithm, which differed in the way that the load set was identified, were compared in terms of their damage detection performance.

Overall, the following conclusions can be drawn at the end of this study: (i) Including only pristine conditions in the training of ANN 1, as was done for version #1 of the algorithm, was sufficient to obtain satisfactory load identification results, while including also damaged conditions, as was done for version #2, did not provide clear benefits. (ii) Recognizing the load set with ANN 1, corresponding to version #1 and version #2 of the algorithm, provided a higher load identification quality compared to using the calibration matrix method, the latter corresponding to version #3 of the algorithm. (iii) The quality of the load recognition step had paramount relevance in the anomaly detection step. Therefore, versions #1 and #2 of the algorithm showed higher anomaly detection performances than version #3. In fact, considering the same PFA, version #1 and #2 provided a higher POD than version #3. (iv) The algorithms have become sensitive to the damage size for a characteristic dimension bigger than 15 mm, and all the algorithm’s versions showed similar performances in the damage assessment and localization step. (v) The effects of damage in the adhesive layer generated very little effect on the strain field, in particular, only the out-of-plane shear strain was influenced.

This study was based on a previous work, which was aimed at assessing the feasibility of an FBG-based SHMS on a helicopter rotor blade, and it demonstrated the feasibility of an SHMS on a real-world component subjected to a complex loading condition. The obtained results provided the procedure for the preliminary design of a strain-based SHMS and allowed for the reduction of the number of blades and tests needed to assess the performance of an SHMS on such a complex structural element.

This work was limited to the hypothesis of model linearity. However, the contact interactions between the blade root and the bearing represented a source of nonlinearity. Future works will be needed to validate the performance of the algorithm with experimental tests, especially to evaluate the effects of the nonlinearity of the problem. Moreover, experiments should be performed by embedding some layers of non-adherent material to replicate a real delamination to assess the modeling of the damage, which in this work was addressed by deleting the cohesive elements of the adhesive layer. A more complex damage scenario should also be investigated, for instance, including damage in the antitorsional layer and/or in the central core. Due to the large number of FBG sensors contributing to a cost increase, a cost–benefit analysis needs to be performed to evaluate the economic impact of the SHMS on the lifecycle of the blade.

## Figures and Tables

**Table 1:** Constrained degrees of freedom, referring to nodes A to L of Figure 4: 1–3 are translations in axes x, y, and z; 4–6 are rotations around axes x, y, and z.

A	B	C	D	E	F	G	H	I	L
1,2,3	1,2,3	2,3	1,2,3	1,2,3	1,2,3	2,3	2,3,4	1,2,3	1,2,3

**Table 2:** The load set for model validation, normalized with respect to BB1.

CF	BB1	CB1	LAT	PL	BB2	CB2
122.27	1	7.5	12.2	3.3	1	−7.66

**Table 3:** Finite Element model validation with experimental data.

Strain Gauge Code	Nonlinear Implicit Model	Linear Model
	Difference % 100 × (Test Values − FEM Strain Values)/Test Value S7	Difference % 100 × (Test Values − FEM Strain Values)/Test Value S7
S1	−26.27	13.77
S2	−1.67	15.95
S3	−3.08	−4.82
S4	−2.05	−11.70
S5	0.17	−8.47
S6	12.2	27.42
S7	10.46	39.62
S8	−14.03	21.46
S9	3.33	6.23
S10	−4.38	10.99
S11	20.46	16.83
S12	5.28	20.99
S13	8.93	31.04
S14	−1.19	13.88

**Table 4:** Artificial Neural Network architectures and training parameters.

ANN Name	Type	Node Number per Layer	Number of Hidden Layers	Training Function	Dataset Partition [Training/Validation/Test]	Activation Function	Input	Target
ANN 1	Regression	10	3	Levenberg–Marquardt backpropagation	[70/15/15]	Hyperbolic tangent sigmoid	Strain at sensing points	Load components
ANN 2	Pattern recognition	10	3	Levenberg–Marquardt backpropagation	[70/15/15]	Hyperbolic tangent sigmoid	Damage indexes at sensing points	Blade’s state of integrity (0 = pristine, 1 = damaged)
ANN 3	Regression	10	2	Levenberg–Marquardt backpropagation	[70/15/15]	Hyperbolic tangent sigmoid	Damage indexes at sensing points	Damage position and damage size

**Table 5:** Load identification methodologies for the three versions of the algorithm.

Algorithm Version	Load Identification Methodology
Version #1	ANN trained only on blade in pristine conditions
Version #2	ANN trained on blade both in pristine and damaged conditions
Version #3	Solution of inverse problem finding load set {*P*} using Equation (3)

**Table 6:** The applied and identified load set in version #1 of the algorithm with 2% Gaussian noise level in the cases of a pristine blade and a damaged blade root. The load set was normalized with respect to BB1.

	CF	BB1	CB1	LAT	PL	BB2	CB2
Applied load set	149.34	1.00	8.27	12.71	3.33	1.00	−8.33
Identified load set Pristine blade root	149.30	1.00	8.30	12.79	3.38	1.01	−8.36
Identified load set Damaged blade root	149.41	1.04	8.31	12.73	3.48	1.04	−8.39

**Table 7:** The real and estimated size and position of the damage for the damaged blade root.

	Damage Size (mm)	Curvilinear Coordinate %	Cross Section Coordinate %
Real values	30	33.33	49.95
Predicted from the algorithm	21.46	30.95	44.75

## Data Availability

The data presented in this study are available on request from the corresponding author due to confidentiality restriction.
